# Carbapenem-Resistant Acinetobacter baumannii in U.S. Hospitals: Diversification of Circulating Lineages and Antimicrobial Resistance

**DOI:** 10.1128/mbio.02759-21

**Published:** 2022-03-21

**Authors:** Alina Iovleva, Mustapha M. Mustapha, Marissa P. Griffith, Lauren Komarow, Courtney Luterbach, Daniel R. Evans, Eric Cober, Sandra S. Richter, Kirsten Rydell, Cesar A. Arias, Jesse T. Jacob, Robert A. Salata, Michael J. Satlin, Darren Wong, Robert A. Bonomo, David van Duin, Vaughn S. Cooper, Daria Van Tyne, Yohei Doi

**Affiliations:** a Division of Infectious Diseases, University of Pittsburgh School of Medicine, Pittsburgh, Pennsylvania, USA; b The Biostatistics Center, The George Washington University, Rockville, Maryland, USA; c Division of Pharmacotherapy and Experimental Therapeutics, University of North Carolina, Chapel Hill, North Carolina, USA; d Department of Infectious Diseases and Microbiology, University of Pittsburgh Graduate School of Public Health, Pittsburgh, Pennsylvania, USA; e Department of Infectious Diseases, Cleveland Clinic, Cleveland, Ohio, USA; f Department of Laboratory Medicine and Pathology, Mayo Clinic, Jacksonville, Florida, USA; g Division of Infectious Diseases, Houston Methodist Hospital, Houston, Texas, USA; h Center for Infectious Diseases Research, Houston Methodist Research Institute, Houston, Texas USA; i Division of Infectious Diseases, Department of Medicine, Emory University School of Medicine, Atlanta, Georgia, USA; j Division of Infectious Diseases and HIV Medicine, Department of Medicine, Case Western Reserve University School of Medicine, Cleveland, Ohio, USA; k Division of Infectious Diseases, Department of Medicine, Weill Cornell Medicine, New York, New York, USA; l Division of Infectious Diseases, Keck School of Medicine of the University of Southern California, Los Angeles, California, USA; m Research Service, Louis Stokes Cleveland Department of Veterans Affairs Medical Center, Cleveland, Ohio, USA; n Departments of Biochemistry, Pharmacology, Molecular Biology and Microbiology, and Proteomics and Bioinformatics, Case Western Reserve University School of Medicine, Cleveland, OH, USA; o CWRU-Cleveland VAMC Center for Antimicrobial Resistance and Epidemiology (Case VA CARES) Cleveland, OH, USA; p Division of Infectious Diseases, University of North Carolina, Chapel Hill, North Carolina, USA; q Department of Microbiology and Molecular Genetics, University of Pittsburgh School of Medicine, Pittsburgh, Pennsylvania, USA; r Departments of Microbiology and Infectious Diseases, Fujita Health University School of Medicine, Aichi, Japan; Carnegie Mellon University

**Keywords:** *Acinetobacter baumannii*, carbapenem resistance, clinical epidemiology, molecular epidemiology

## Abstract

Carbapenem-resistant Acinetobacter baumannii (CR*Ab*) is a major cause of health care-associated infections. CR*Ab* is typically multidrug resistant, and infection is difficult to treat. Despite the urgent threat that CR*Ab* poses, few systematic studies of CR*Ab* clinical and molecular epidemiology have been conducted. The Study Network of Acinetobacter as a Carbapenem-Resistant Pathogen (SNAP) is designed to investigate the clinical characteristics and contemporary population structure of CR*Ab* circulating in U.S. hospital systems using whole-genome sequencing (WGS). Analysis of the initial 120 SNAP patients from four U.S. centers revealed that CR*Ab* remains a significant threat to hospitalized patients, affecting the most vulnerable patients and resulting in 24% all-cause 30-day mortality. The majority of currently circulating isolates belonged to ST2^Pas^, a part of clonal complex 2 (CC2), which is the dominant drug-resistant lineage in the United States and Europe. We identified three distinct sublineages within CC2, which differed in their antibiotic resistance phenotypes and geographic distribution. Most concerning, colistin resistance (38%) and cefiderocol resistance (10%) were common within CC2 sublineage C (CC2C), where the majority of isolates belonged to ST2^Pas^/ST281^Ox^. Additionally, we identified ST499^Pas^ as the most common non-CC2 lineage in our study. Our findings suggest a shift within the CR*Ab* population in the United States during the past 10 years and emphasize the importance of real-time surveillance and molecular epidemiology in studying CR*Ab* dissemination and clinical impact.

## INTRODUCTION

Carbapenem-resistant Acinetobacter baumannii (CR*Ab*) constitutes a major threat to public health. CR*Ab* isolates are extensively resistant to multiple antimicrobial agents, are often spread among hospitalized patients, and cause difficult-to-treat infections associated with high mortality (1). The World Health Organization (WHO) and the Centers for Disease Control and Prevention (CDC) have designated CR*Ab* a priority pathogen based on the lack of effective treatment options and have pointed to an urgent need for additional research (2–4). Prior studies have shown that several genetically distinct clonal A. baumannii lineages/groups are currently circulating around the world, with the three most prevalent global lineages referred to as clonal complex 1 (CC1), CC2, and CC3. These designations are reflected in the multilocus sequence types of each lineage (ST1, ST2, and ST3, respectively) as defined by the commonly used Pasteur Institute scheme. ST2^Pas^ is the most common CC2 lineage in the United States, and other CC2 and non-CC2 lineages are found less commonly (5, 6). Our previous analysis at a single health system in Pennsylvania found substantial genetic diversity within extensively drug-resistant ST2^Pas^ A. baumannii, which could be grouped into multiple distinct sublineages by multilocus sequence typing (MLST) and whole genome sequencing (WGS) analysis (7). Despite its being a major public health concern, our current understanding of the CR*Ab* lineages and sublineages circulating in the United States is limited. Systematic studies of CR*Ab* are of paramount importance in devising strategies to prevent their dissemination and improve clinical outcomes.

The Study Network of Acinetobacter as a Carbapenem-Resistant Pathogen (SNAP) is a prospective, observational, multicenter clinical study that is designed to elucidate the clinical characteristics, treatment outcomes, and contemporary genomic epidemiology of CR*Ab* through consecutive enrollment of hospitalized patients with clinical cultures positive for CR*Ab* at multiple health systems throughout the United States. In this analysis, we describe the results from this effort, comprising 120 unique patients and 150 CR*Ab* isolates collected during the first year of the study from four health care systems in the United States, with a focus on patient characteristics, bacterial population structure, and antibiotic resistance profiles.

## RESULTS

### Patients and clinical epidemiology.

One hundred twenty unique patients admitted to twenty three hospitals at four health systems in the United States in 2017 and 2018 were enrolled in the first phase of the SNAP cohort (Table 1). During the period of enrollment, none of the study sites identified CR*Ab* outbreaks. In this cohort, 135 admissions were recorded, and 155 clinical cultures yielded CR*Ab* (1 to 5 isolates per patient). Clinical data were available from all 120 patients. The enrollments were from Cleveland, OH (55%), Pittsburgh, PA (23%), Houston, TX (20%), and Chapel Hill, NC (3%) area hospitals. Median patient age was 61, and 60% were male. Most patients had comorbid conditions, with a median Charlson comorbidity score of 3, and 48% were critically ill (Pitt bacteremia score, ≥4) at the time of initial CR*Ab* isolation (8). More than half of patients were admitted from long-term care settings, with 41% admitted from long-term chronic care facilities (nursing homes) and an additional 11% from long-term acute-care hospitals. Information on whether patients received ventilator or tracheostomy care prior to admission was not available. All-cause mortality rates at 30 and 90 days from the date of index culture collection were 24% and 27%, respectively. Thirty- and 90-day mortality in those deemed to have infection was 26%. Readmission within 90 days occurred in 54% of cases. Using DOOR (desirability of outcome ranking) outcomes at 30 days after index culture, 44% were alive without events, 19% were alive with one event, and 13% were alive with two or three events.

**TABLE 1:** Clinical characteristics and outcomes following collection of the index CR*Ab* isolate from each patient

Characteristic	Value (*n* = 120)^*a*^
Median age at culture (IQR^*b*^)	61 (51–70)
Gender	
Male	72 (60)
Female	48 (40)
Race	
White	74 (62)
Black	33 (28)
Other	7 (6)
Unknown	6 (5)
Median CCI (IQR)^*c*^	3 (1–4)
Median Pitt bacteremia score (IQR)^*d*^	3 (2–6)
Admission type	
Admission from home	37 (31)
Transfer from other hospital	21 (18)
Transfer from long-term chronic care facility	49 (41)
Transfer from long-term acute care	13 (11)
Study site	
Cleveland Clinic Foundation	66 (55)
University of North Carolina at Chapel Hill	3 (3)
University of Pittsburgh Medical Center	27 (23)
University of Texas Hospitals	24 (20)
Culture	
Respiratory infection	33 (28)
Respiratory colonization	23 (19)
Wound infection	20 (17)
Wound colonization	23 (19)
Blood infection	9 (8)
Urine infection	3 (3)
Urine colonization	6 (5)
Other colonization	2 (2)
Nonwound abdominal infection	1 (1)
DOOR category at 30 days^*e*^	
Alive without events	53 (44)
Aline with one event	23 (19)
Alive with two or three events	15 (13)
Dead	29 (24)
DOOR event at 30 days	
Dead or discharged to hospice	24 (20)
No clinical response	41 (34)
Renal failure	8 (7)
C. difficile infection	3 (3)
Mortality	
30 days	29 (24)
90 days	32 (27)
Mortality among subjects with infection	
30 days	17 (26)
90 days	17 (26)
Readmission at 90 days	51 (54)
Readmission at 90 days among subjects with infection	25 (38)

a Data are number (%) unless stated otherwise.

b IQR, interquartile range.

c Charlson comorbidity index (CCI) is a chronic comorbidity score with a range from 0 to 37, with higher scores indicating more comorbid conditions present. A patient with a score of 3 could have three level 1 comorbid conditions (e.g., dementia, chronic pulmonary disease, and congestive heart failure), one level 1 condition (e.g., dementia) and one level 2 condition (e.g., leukemia), or one level 3 condition (moderate or severe liver disease).

d Pitt bacteremia score is an acute severity of illness score. Higher scores indicate more severe illness. A patient with a score of 3 would have one level 1 marker of acute illness (e.g., disoriented mental state) and one level 2 marker (e.g., hypotension).

e DOOR (desirability of outcome ranking) analysis components are defined in Materials and Methods.

Among the 120 enrolled patients, the respiratory tract was the predominant anatomic source of the first available CR*Ab* isolate (47%), followed by wounds (36%) (Table 1). Fifty-nine percent of isolates from respiratory source were associated with infection, while 46% of isolates from wound cultures and 33% from urine cultures met the definition of infection.

### Molecular epidemiology and bacterial population structure.

To understand the population structure and distribution of CR*Ab* in the United States, phylogenetic analyses and multilocus sequence type (MLST) identification were performed on the first available isolate from each patient.

**FIG 1:**
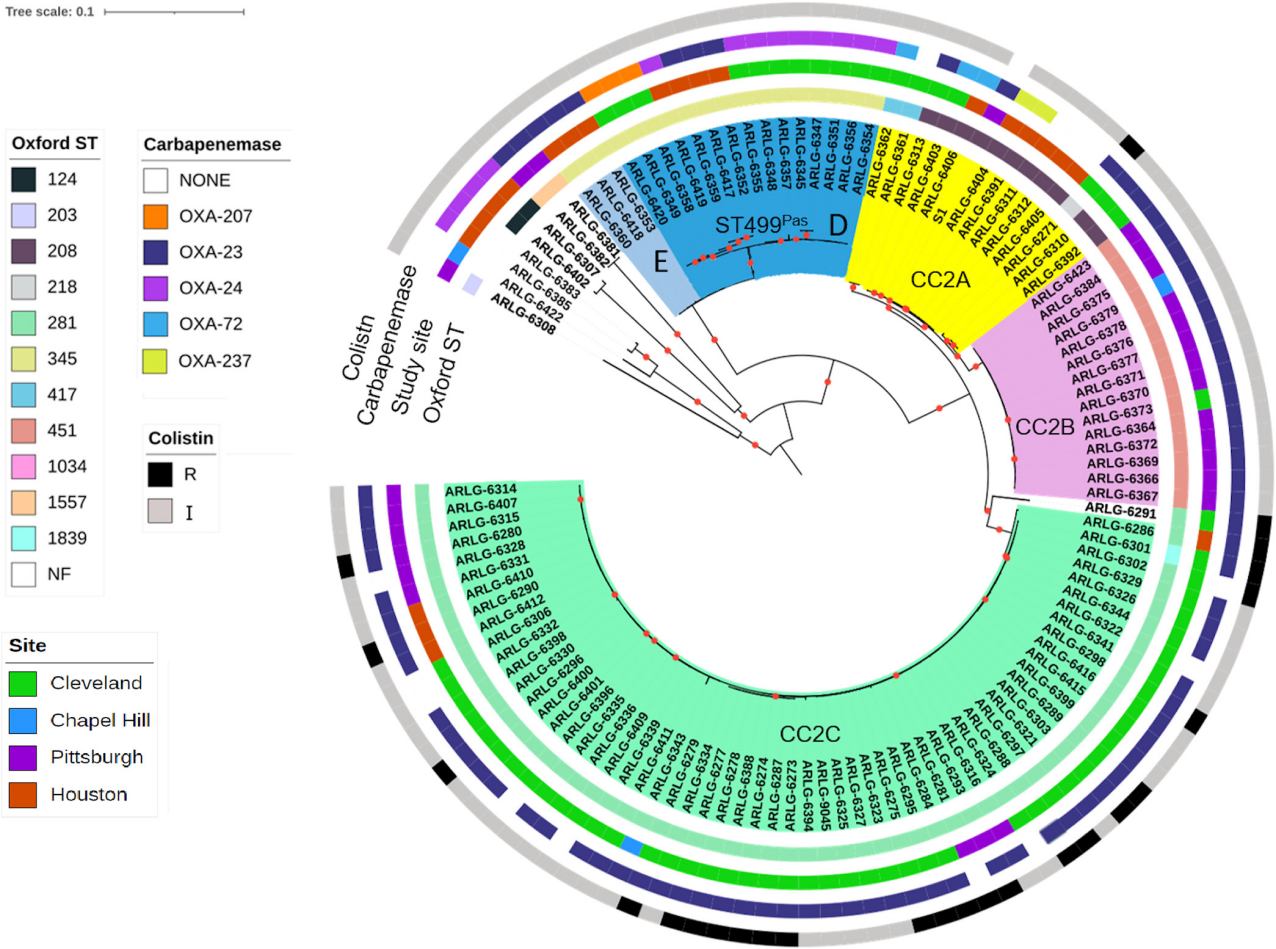
Core genome phylogeny of 115 CR*Ab* isolates from four medical centers in the United States. The first isolate sampled from each patient was included, and the midpoint rooted phylogeny was constructed from SNPs detected in the core genomes of all isolates (2.6-Mb core genome length), using RAxML. The phylogeny is annotated based on Oxford ST and study center of isolation. Branches are shaded by lineages and sublineages described in the text. Nodes supported by bootstrap values of 100 are marked with red dots. NF, not found; R, resistant; I, intermediate. An interactive version of this figure is available online at http://arlg.med.unc.edu/crackle/.

A whole-genome phylogeny showed a diverse population structure (Fig. 1). We defined single-nucleotide-polymorphism (SNP) thresholds based on the observed SNP distribution to cluster study isolates into clearly separated clonal lineages and sublineages, which were then compared to established Pasteur (Pas) and Oxford (Ox) MLST schemes. A cutoff of 10,000 SNPs differentiated CR*Ab* lineages belonging to different Pasteur ST types and CCs. A cutoff 2,000 SNPs further defined major sublineages within the Pasteur STs, which largely correlated with Oxford STs. The 115 available isolates belonged to 10 different Pasteur sequence types (STs), including 2 novel STs first reported by this study (ST1562^Pas^ and ST1563^Pas^). The majority of isolates (77%) belonged to ST2^Pas^ or CC2, the dominant antibiotic-resistant lineage that has circulated in the United States and Europe (5). Three sublineages within CC2 with various degrees of heterogeneity were apparent (Fig. 1; Table S1). CC2 sublineage A (CC2A) comprised multiple Oxford STs, including ST208^Ox^, ST218^Ox^, and ST417^Ox^ (Tables S1 and S2). CC2 sublineages B (CC2B) and C (CC2C) corresponded to ST451^Ox^ and its single locus variants (SLVs) and to ST281^Ox^ and its SLVs, respectively (Table S2).

10.1128/mBio.02759-21.2 TABLE S1 Pairwise core genome SNP comparisons calculated from total core genome of 150 isolates from 120 patients included in the study. Download Table S1, DOCX file, 0.01 MB. Copyright © 2022 Iovleva et al. 2022 Iovleva et al. https://creativecommons.org/licenses/by/4.0/ This content is distributed under the terms of the Creative Commons Attribution 4.0 International license.

10.1128/mBio.02759-21.3 TABLE S2 Pairwise core genome SNP comparisons among major sublineages calculated from total core genomes of 150 CR*Ab* isolates from 120 study patients. Download Table S2, DOCX file, 0.01 MB. Copyright © 2022 Iovleva et al. 2022 Iovleva et al. https://creativecommons.org/licenses/by/4.0/ This content is distributed under the terms of the Creative Commons Attribution 4.0 International license.

Of the remaining isolates, most belonged to ST499^Pas^ (16%). The non-CC2 ST499^Pas^ lineage could be further separated into two sublineages, D and E (containing 17 and 3 isolates, respectively), with both sublineages corresponding to the same Oxford ST (ST345^Ox^). The remaining isolates belonged to 7 additional STs and contained one or two isolates each.

The sublineage that is widely distributed in the United States, corresponding to ST208^Ox^ and here called sublineage CC2A, was found only in Cleveland and Houston (Table 2). Isolates belonging to sublineage CC2B (ST451^Ox^ and its SLVs) were predominantly found in Pittsburgh but were also detected in Cleveland and Chapel Hill. Sublineage CC2C (ST281^Ox^ and SLVs) was found at all four study centers and was the dominant lineage in Cleveland and Pittsburgh. ST499^Pas^ lineage D isolates were identified in Cleveland and Houston, while lineage E isolates were found only in Houston (Table 2). At the level of individual hospitals, some had a single dominant lineage, while others had several dominant lineages. The distributions of CR*Ab* sublineages from different body sites were similar to one other, with the exception that no CC2A isolates were found in blood cultures and no CC2B isolates were found in wound cultures (Table 3).

**TABLE 2:** Geographic distribution of CR*Ab* isolates by study site

Sublineage	No. (%)				
	Total (*n* = 115)	Cleveland (*n* = 66)	Pittsburgh (*n* = 25)	Houston (*n* = 21)	Chapel Hill (*n* = 3)
CC2A	13 (11)	7 (11)	None	6 (29)	None
CC2B	15 (13)	1 (2)	13 (52)	None	1 (33)
CC2C	60 (52)	46 (70)	9 (36)	4 (19)	1 (33)
ST499^Pas^	18 (16)	11 (17)	None	7 (33)	None
Other ST	9 (8)	1 (2)	3 (12)	4 (19)	1 (33)

**TABLE 3:** Culture source distribution of CR*Ab* isolates

Sublineage	No. (%)						
	Total (*n* = 115)	Respiratory (*n* = 53)	Wound (*n* = 40)	Blood (*n* = 8)	Urine (*n* = 8)	Other (*n* = 5)	NA^*a*^ (*n* = 1)
CC2A	13 (11)	5 (38)	6 (46)	0 (0)	1 (8)	1 (8)	None
CC2B	15 (13)	10 (67)	0 (0)	2 (13)	1 (7)	2 (13)	None
CC2C	60 (52)	27 (45)	25 (42)	2 (3)	3 (5)	2 (5)	1 (2)
ST499^Pas^	18 (16)	6 (33)	7 (39)	3 (16)	2 (11)	0 (11)	None
Other ST	9 (8)	5 (56)	2 (22)	1 (11)	1 (11)	0	None

a NA, not available.

### Antibiotic susceptibility of CR*Ab* isolates.

**FIG 2:**
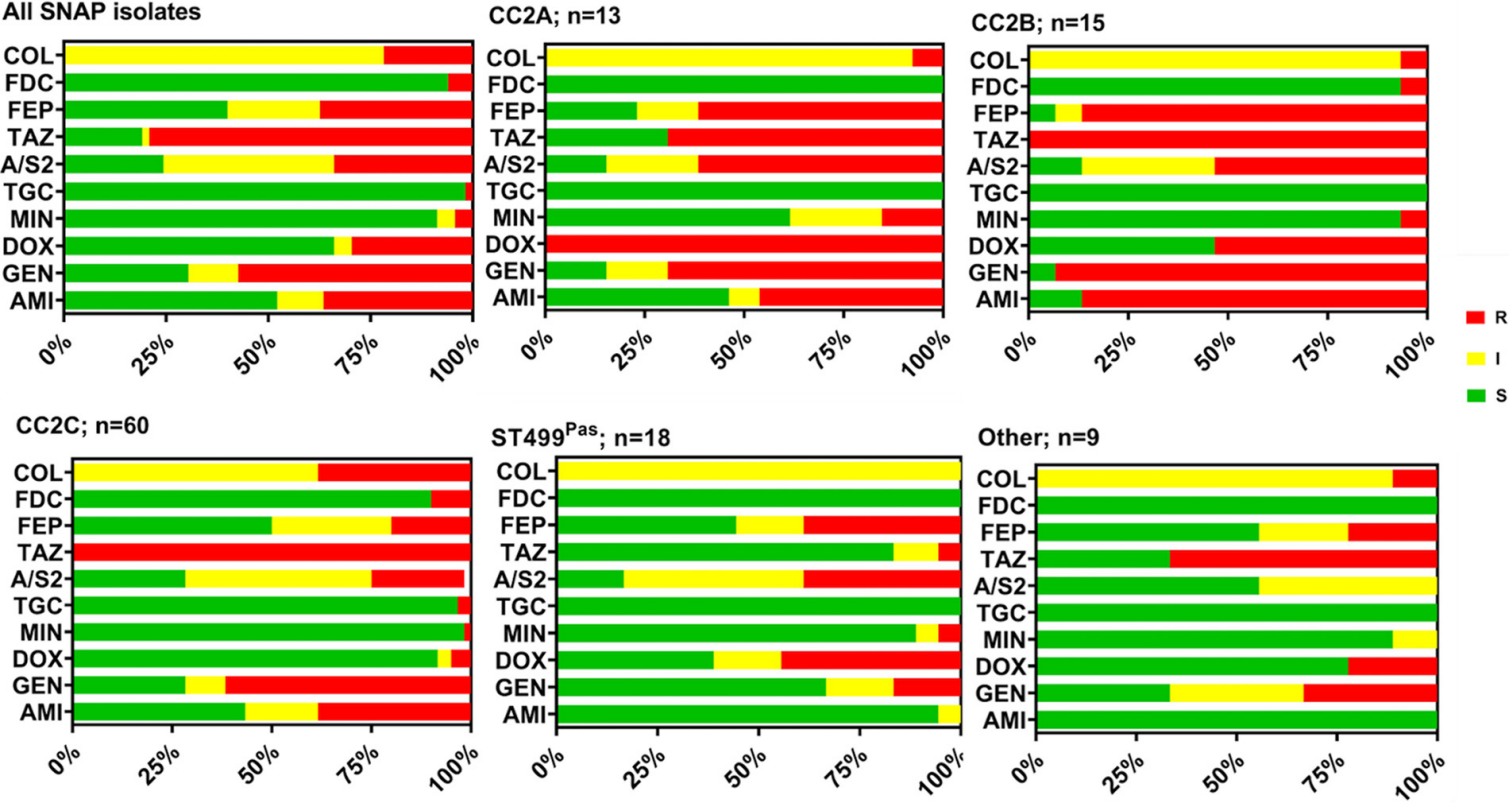
Antimicrobial susceptibility profiles of CR*Ab* isolates. Antimicrobial susceptibilities of 115 initial patient isolates were determined with Sensititre plates or broth microdilution. COL, colistin; FDC, cefiderocol; FEP, cefepime; TAZ, ceftazidime; A/S2, ampicillin-sulbactam; TGC, tigecycline; MIN, minocycline; DOX, doxycycline; GEN, gentamicin; AMI, amikacin. S, susceptible; I, intermediate; R, resistant. Susceptibilities were assigned according to CLSI guidelines.

We performed MIC testing on the 115 index isolates from unique patients for agents that possess activity against A. baumannii. Of the 115 isolates tested, 36% were resistant to amikacin and 57% were resistant to gentamicin (Fig. 2; Table S3). Rates of tigecycline and minocycline resistance were low at 2% and 4%, respectively, whereas 37% of isolates were resistant to cefepime, and 79% were resistant to ceftazidime. Seven isolates (6%) were resistant to cefiderocol using CLSI criteria (MIC, ≥16 μg/mL). Finally, 22% of isolates in our study were resistant to colistin, the last-resort antibiotic for treating CR*Ab* infections. Due to the recent change of colistin breakpoints by CLSI, the remaining isolates were intermediate to colistin, eliminating the susceptible category and moving susceptible to intermediate. When we assessed antibiotic susceptibility rates by bacterial sublineage, a few notable trends emerged. CC2B isolates had the highest aminoglycoside and cefepime nonsusceptibility rates compared to other lineages. All but two (13/15 [87%]) CC2B isolates were resistant to amikacin compared to 42% (42/100) of isolates from other lineages (*P *= 0.0012). Similarly, 93% (14/15) of CC2B isolates were resistant to gentamicin, compared to 66% from other lineages (*P* = 0.036). Finally, 93% (14/15) of CC2B isolates were resistant to cefepime, compared to 54% (54/100) from the other sublineages (*P *= 0.004 by chi square test). CC2C isolates were more likely to be resistant to colistin (23/60 [38%]) than others (3/55 [5%]) (*P* < 0.0001). Study sites differed somewhat in the proportion of colistin-resistant CC2C isolates: 38% in Cleveland, 33% in Pittsburgh, and 50% in Houston. Colistin susceptibility differences between lineages persisted in Cleveland and Houston (*P *< 0.05 for both locations). Six of seven cefiderocol-resistant isolates belonged to CC2C, and one of these isolates was also resistant to colistin (6/60 [10%]). Compared to other lineages, ST499^Pas^ (D and E) isolates had low rates of nonsusceptibility to ceftazidime (3/18 [17%] versus 90/97 [93%]; *P *< 0.0001) but not cefepime (10/18 [56%] versus 58/97 [60%]; *P *= 0.74). However, since cephalosporins are not traditionally used to treat CR*Ab*, we do not currently have any clinical outcome data that would help establish the potential utility of ceftazidime in this case. Overall, these data suggest that the genetic background and evolutionary history of sublineages might affect CR*Ab*’s susceptibility to antimicrobial therapy.

10.1128/mBio.02759-21.4 TABLE S3 Antimicrobial susceptibility profiles of CR*Ab* isolates. Antimicrobial susceptibilities of 115 initial patient isolates were determined with Sensititre plates or by broth microdilution. COL, colistin; FDC, cefiderocol; FEP, cefepime; TAZ, ceftazidime; A/S2, ampicillin-sulbactam; TGC, tigecycline; MIN, minocycline; DOX, doxycycline; GEN, gentamicin; AMI, amikacin. S, susceptible; I, intermediate; R, resistant. Susceptibilities were assigned according to CLSI guidelines. Tigecycline susceptibility was assigned based on FDA *Enterobacterales* breakpoints. Download Table S3, DOCX file, 0.02 MB. Copyright © 2022 Iovleva et al. 2022 Iovleva et al. https://creativecommons.org/licenses/by/4.0/ This content is distributed under the terms of the Creative Commons Attribution 4.0 International license.

### Plasmids and resistance islands.

One of the reasons for the success of CR*Ab* as a nosocomial pathogen is its ability to acquire drug resistance genes through horizontal transfer. In addition to the ability to acquire plasmids, CR*Ab* isolates are also known to possess composite transposons and integrons containing resistance genes at chromosomal locations, referred to as resistance islands (RIs) (9, 10).

To determine plasmid diversity within clinical CR*Ab* isolates, sequences of six unique plasmids were resolved from available high-quality, closed genomes of isolates belonging to CC2A (S1), CC2B (ARLG-6376), CC2C (ARLG-6295 and ARLG-6344), ST499^Pas^ D (ARLG-6420), and ST499^Pas^ E (ARLG-6418) (Table S4). Plasmids varied in size from 11 kb to 167 kb and belonged to five different homology groups based on *rep* gene sequences. Three of the plasmids carried OXA-type carbapenemase genes (*bla*_OXA-23_ in pARLG-6295_2 and pARLG-6344_2; *bla*_OXA-207_ in pARLG-6420_2). pARLG-6295_2 additionally carried the *aphA6* gene, conferring amikacin resistance. The remaining two plasmids did not possess known antimicrobial resistance genes.

10.1128/mBio.02759-21.5 TABLE S4 CR*Ab* plasmid characteristics. Download Table S4, DOCX file, 0.01 MB. Copyright © 2022 Iovleva et al. 2022 Iovleva et al. https://creativecommons.org/licenses/by/4.0/ This content is distributed under the terms of the Creative Commons Attribution 4.0 International license.

**FIG 3:**
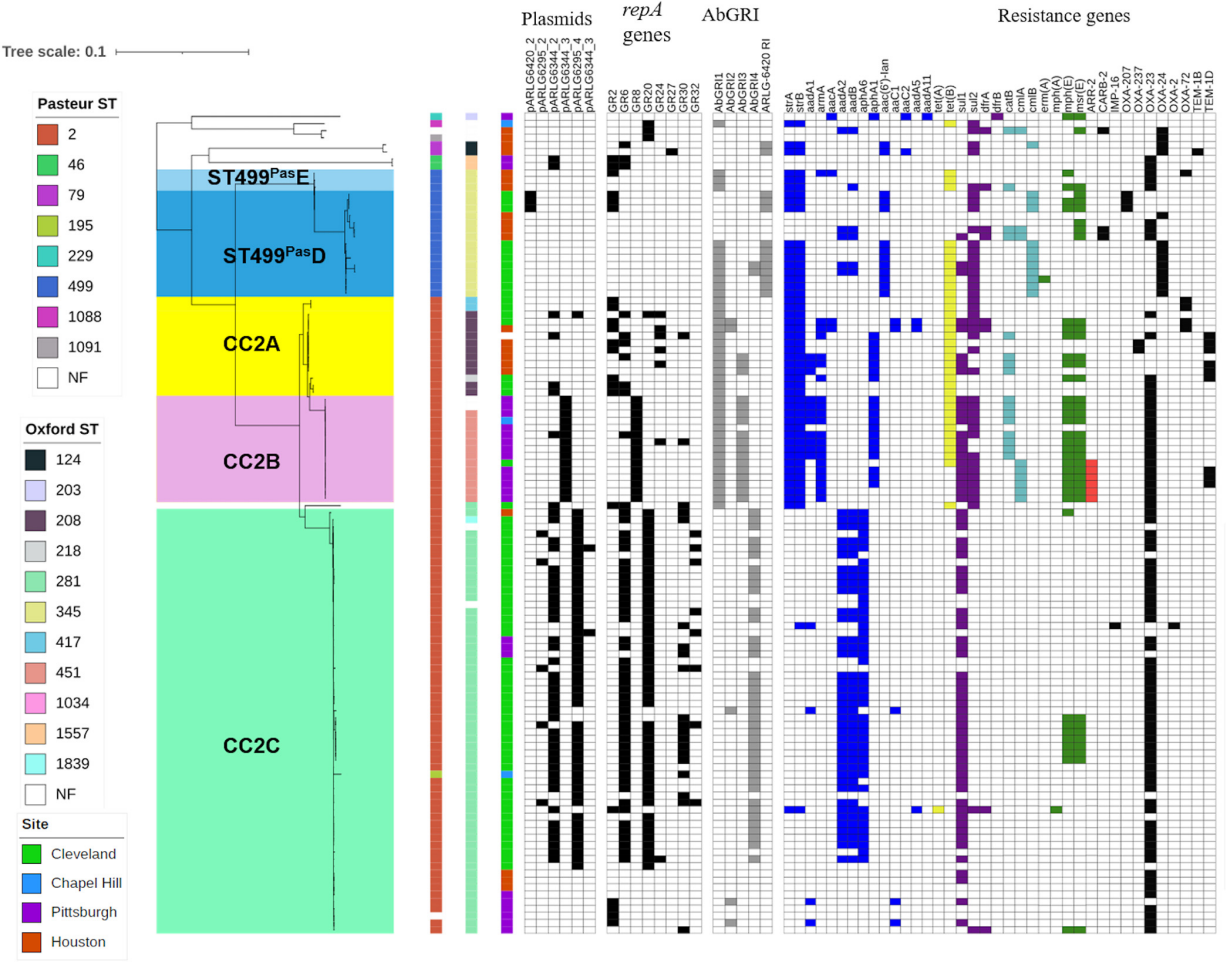
CR*Ab* phylogenetic tree from Fig. 1 shown with plasmid content, genomic resistance islands (AbGRI), and antibiotic resistance genes. Plasmids were identified by long-read sequencing and *repA* gene sequence presence. Presence of genetic elements is notated with color boxes; absence is shown with white boxes. Plasmid and β-lactamase gene presence is shown with black boxes. AbGRI presence is shown with gray boxes. Resistance gene presence is shown with colored boxes: blue, aminoglycoside resistance; yellow, tetracycline resistance; purple, sulfonamide resistance; turquoise, chloramphenicol resistance; green, macrolide resistance; red, rifampin resistance.

Next, we evaluated the presence of identified plasmids among all initial CR*Ab* isolates in our study (Fig. 3). Overall, CC2 isolates carried significantly more plasmids than non-CC2 isolates (*P* < 0.0001). Within CC2, different sublineages had differences in plasmid content, where most CC2B isolates contained pARLG-6344_3 while CC2C tended to harbor the *bla*_OXA-23_-carrying plasmids pARLG-6344_2 and pARLG-6295_4. CC2C isolates that did not contain pARLG-6344_2 had either the *bla*_OXA-23_- and *aphA6*-carrying plasmid pARLG-6295_2 or no detectable carbapenemase gene-carrying plasmids. The majority of ST499^Pas^ isolates lacked plasmids, with the exception of three isolates carrying *bla*_OXA-207_ on pARLG-6420_2. To account for additional plasmids not identified through analysis of hybrid-assembled genomes, we also examined the sequences for the presence of plasmid *rep* genes (9). Overall, plasmid *rep* genes belonging to eight groups were identified among all initial CR*Ab* isolates. Plasmid *rep* gene content was also higher in CC2 than other lineages (*P* < 0.0001).

We then surveyed the isolates for the presence of previously described RIs, including AbGRI1, AbGRI2, AbGRI3, and AbGRI4 (Fig. 3) (11). Most CC2A and CC2B isolates, along with some ST499^Pas^ isolates, possessed an AbGRI1-like island, which typically carries *strA-strB* (streptomycin resistance), *tetA*(B) (tetracycline resistance), and *bla*_OXA-23_ genes. Most CC2B and several CC2A isolates belonging to ST2^Pas^/ST208^Ox^ also contained an AbGRI3-like RI carrying *aacA4* (gentamicin/tobramycin resistance), *catB8* (chloramphenicol resistance), *aadA1* (streptomycin resistance), and *armA* (gentamicin, kanamycin, amikacin, tobramycin, and plazomicin resistance). CC2C isolates almost exclusively contained the recently described AbGRI4 island containing *aadB* (tobramycin resistance), *aadA2* (streptomycin and spectinomycin resistance), and *sul1* (sulfonamide resistance) genes. A small group of CC2C isolates lacked AbGRI4 and contained either an AbGRI2-like RI, which typically carried *aacC1* (gentamicin resistance), *aadA1* (streptomycin resistance), and *sul1* (sulfonamide resistance), or no RIs at all.

**FIG 4:**
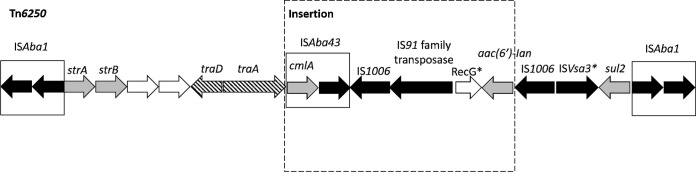
Tn*6250*-like resistance island identified in ST499^Pas^ and ST79^Pas^ isolates. Nomenclature and labeling match the original publication of LAC-4 genome for comparison. Direction of transcription of genes is shown by arrows. Dark arrows denote transposase genes. Gray arrows denote antimicrobial resistance genes. Striped arrows and open arrows denote genes involved in conjugation and genes with unknown function, respectively. An asterisk indicates the partial nature of the element.

Additionally, we identified an RI that was exclusively present in ST499^Pas^ and ST79^Pas^ isolates within our data set. This RI (ARLG-6420 RI) was 19.5 kb long and was integrated at the tRNA-Ser site. It possessed 99.8% sequence identity with Tn*6250* and an additional insertion of an IS*Aba43*-IS*1006*-IS*91*-Δ*recG*-*aac(6′)-Ian* element (Fig. 4) (12, 13). While the plasmids resolved in our study appeared to carry relatively few resistance genes, most of these genes were for OXA carbapenemases, which eliminate carbapenems from the therapeutic armamentarium. Resistance to other agents was likely due to a combination of RI-encoded acquired antimicrobial resistance genes and mutations and regulatory changes in intrinsic resistance-associated genes. Although our analysis of plasmid and RI content was not comprehensive given the limited number of closed genomes available, it provides an initial assessment of the diversity of plasmids and RIs found among contemporary CR*Ab* isolates in the United States. Both plasmids and resistance islands were abundant among CR*Ab* isolates, and they encoded clinically relevant antimicrobial resistance genes that likely contributed to the persistence of CR*Ab* in clinical settings.

### Carbapenem resistance mechanisms.

We catalogued all genomes for carbapenemase genes that would explain their CR*Ab* phenotype. The acquired carbapenemase gene detected most frequently was *bla*_OXA-23_, which was present in 69% of the isolates (Fig. 3). Other acquired *bla*_OXA_ carbapenemase genes were identified less frequently and included *bla*_OXA-24/40_, *bla*_OXA-72_ and *bla*_OXA-207_ (encoding single-amino-acid variants of OXA-24/40), and *bla*_OXA-237_ (encoding a recently characterized OXA-235-like carbapenemase) (14–16). Fourteen isolates belonging to different sublineages, primarily CC2A and CC2C, did not carry known acquired carbapenemase genes, despite being resistant to carbapenems. Of these 14 isolates, 8 isolates possessed an insertion of IS*Aba1*, an insertion sequence carrying strong promoter activity upstream of the intrinsic carbapenemase gene *bla*_OXA-82_ whose product shows weak carbapenemase activity at baseline expression. The same IS*Aba1* insertion upstream of *bla*_OXA-82_ was previously reported to result in carbapenem resistance in A. baumannii (17). Another 4 isolates possessed IS*Aba1* insertions upstream of other chromosomal carbapenemase genes, including *bla*_OXA-172_, *bla*_OXA-113_, and *bla*_OXA-916_.

### Colistin resistance in CC2C.

Given the surprisingly high rate of colistin resistance in CC2C isolates, we explored possible mechanisms of colistin resistance within this sublineage. None of the isolates contained *mcr* gene family sequences encoding acquired colistin resistance determinants (18). Additionally, we examined the sequence of the *pmrCAB* operon, which is responsible for lipopolysaccharide (LPS) modifications leading to colistin resistance, as well as the *lpxA*, *lpxC*, and *lpxD* genes, which are involved in LPS synthesis and whose disruption can result in colistin resistance (19, 20). We found that *pmrA*, *lpxA*, and *lpxC* had identical nucleotide sequences among both colistin-intermediate and colistin-resistant isolates. Nonsynonymous *pmrB* mutations were present in 30% of colistin isolates (L9P, I25F, M145K, F155V, E185K, F387Y, and N353S). We also identified a D95E substitution in *lpxD* in one isolate. The contribution of *pmrC* and *eptA* sequence variation could not be evaluated. For 65% of the isolates, we could not identify a mechanism of colistin resistance based on the analysis of candidate resistance-associated genes.

### Frequency of recombination events.

**FIG 5:**
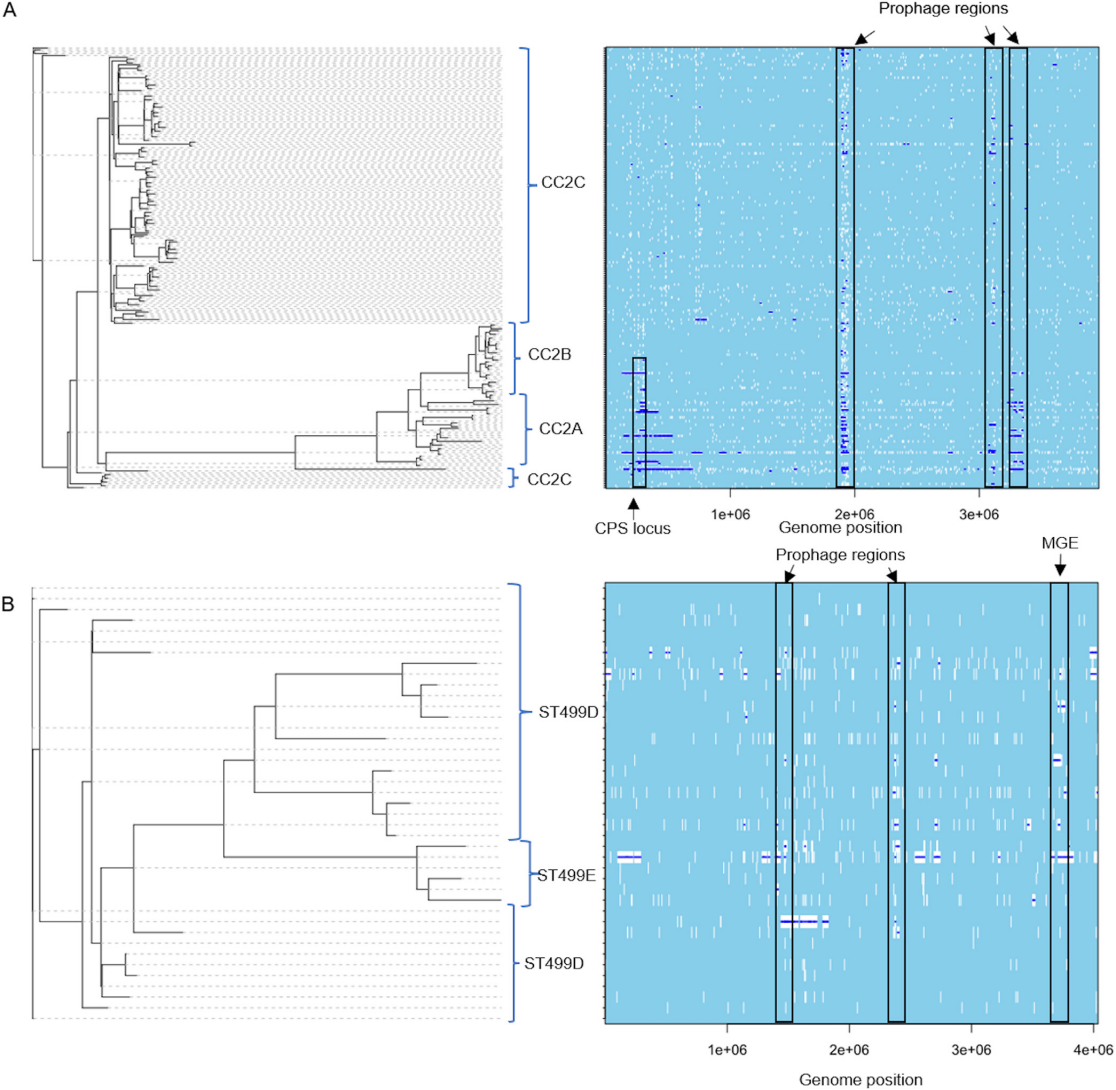
ClonalFrameML analysis of recombination in CR*Ab* lineages CC2 (A) and ST499^Pas^ (B). White vertical bars represent nucleotide substitutions along each branch of the phylogenetic tree. Dark blue horizontal bars indicate putative recombination events. Major CC2 lineages are identified by blue brackets. Recombination hot spots are identified with black rectangles. The S1 genome was used as a reference for the CC2 lineage, while the ARLG-6345 closed genome was used for the ST499^Pas^ lineage. CPS, capsular polysaccharide.

An important question in bacterial population genetics is the extent to which recombination contributes to or constrains lineage diversity. We used ClonalFrameML to analyze all 150 available CR*Ab* genomes (Fig. 5A and B) and discovered an overall recombination event rate of 64 for every 100 point mutations. Within CC2 and ST499^Pas^, the rates were 43 and 49 per 100 point mutations, respectively. Major recombination hot spots within CC2 occurred in probable prophage regions and in the capsular polysaccharide locus (Fig. 5A). Several other long putative recombination events distinguished different sublineages and Oxford STs within CC2 and affected predicted capsular polysaccharide loci and surrounding genes, including *gpi*, which is one of the genes involved in defining Oxford ST (Fig. 5A) (21). Similarly, within ST499^Pas^, larger recombination hot spots occurred in predicted prophage regions, as well as in other putative mobile genomic elements (MGEs), including the Tn*6250*-like genomic island described above. However, recombination events were spread out throughout the genome, largely sparing the capsular polysaccharide (CPS) locus in this clade (Fig. 5B). Finally, removal of recombinant SNPs decreased the number of SNPs in the core genomes and merged the CC2A and CC2B sublineages and the ST499^Pas^ D and ST499^Pas^ E lineages (Tables S6 and S7). These data demonstrate high rates of recombination within CR*Ab* populations that led to differentiation of CR*Ab* sublineages within both CC2 and ST499^Pas^.

10.1128/mBio.02759-21.7 TABLE S6 Post-ClonalFrameML pairwise core genome SNP comparisons calculated from total core genomes of 150 isolates from 120 patients included in the study. Download Table S6, DOCX file, 0.01 MB. Copyright © 2022 Iovleva et al. 2022 Iovleva et al. https://creativecommons.org/licenses/by/4.0/ This content is distributed under the terms of the Creative Commons Attribution 4.0 International license.

10.1128/mBio.02759-21.8 TABLE S7 Post-ClonalFrameML pairwise core genome SNP comparisons among major sublineages calculated from total core genomes of 150 CR*Ab* isolates from 120 study patients. Download Table S7, DOCX file, 0.01 MB. Copyright © 2022 Iovleva et al. 2022 Iovleva et al. https://creativecommons.org/licenses/by/4.0/ This content is distributed under the terms of the Creative Commons Attribution 4.0 International license.

### Intra- and interpatient genetic diversity.

We next assessed the genetic diversity of the CR*Ab* isolates within and between patients. Of the 24 patients with more than one isolate from different culture dates, 22 yielded CR*Ab* isolates belonging to the same Oxford ST. The two remaining patients had CR*Ab* isolates belonging to two distinct Oxford STs, suggesting that most patients with multiple isolates were colonized or infected with the same bacterial strain over time, rather than multiple genetically unrelated strains.

**FIG 6:**
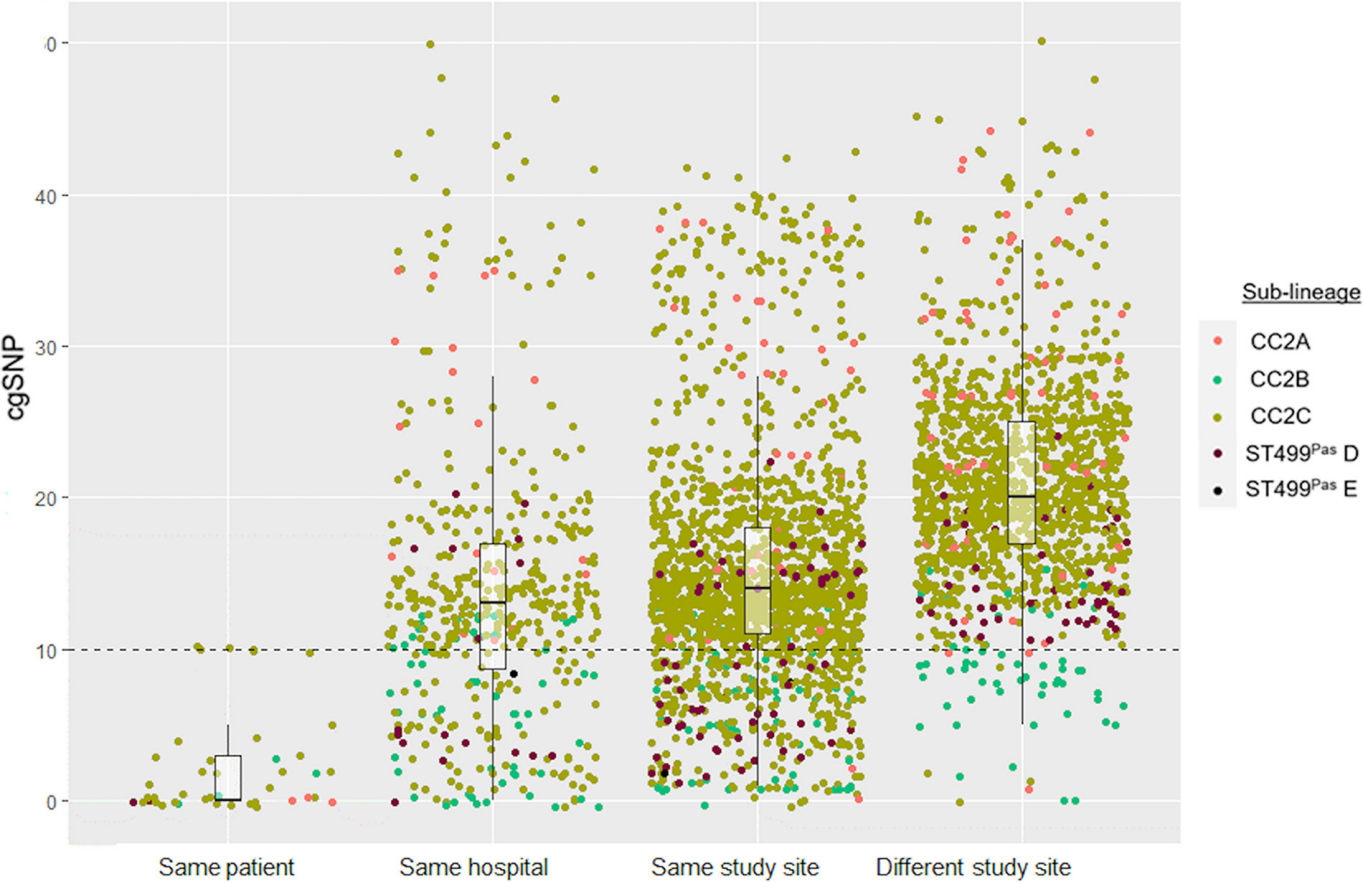
Pairwise nonrecombinant core genome SNP distance comparisons between CR*Ab* isolates of the same sublineage. A total of 150 isolates from 120 patients were included, and comparisons are color coded by sublineage. Box plots indicate median (horizontal line), interquartile range (box edges), and 1.5× the interquartile range (whiskers) for each group. The dashed horizontal line marks a SNP distance of 10, which corresponds to the maximum pairwise SNP difference observed between isolates sampled from the same patient.

Overall, isolates belonging to the same sublineage derived from the same patient had a median pairwise nonrecombinant SNP distance of 0 (range, 0 to 10), with 66% of the genome considered core for SNP calling (Fig. 6; Table S5). Within the same-patient group, CC2A, CC2B, and ST499^Pas^D isolates collected from the same patients were very closely related (range, 0 to 3 SNPs), while CC2C isolates tended to have more SNPs in pairwise comparisons (range, 1 to 10 SNPs). Higher pairwise SNP differences were observed among isolates from the same hospital, from the same study site, and from different study sites for all sublineages than same-patient isolates. Based on these data, we conclude that isolates that are up to approximately 10 SNPs apart likely belong to the same strain, and SNP differences this small between isolates from different patients may indicate recent transmission. However, we cannot infer definitive intra- and interhospital transmission events, as further epidemiologic data are not available.

10.1128/mBio.02759-21.6 TABLE S5 Sublineage-specific pairwise nonrecombinant SNP comparisons between isolates sampled from the same patient, the same study site, and different study sites calculated from total core genomes of 150 CR*Ab* isolates from 120 patients. Download Table S5, DOCX file, 0.01 MB. Copyright © 2022 Iovleva et al. 2022 Iovleva et al. https://creativecommons.org/licenses/by/4.0/ This content is distributed under the terms of the Creative Commons Attribution 4.0 International license.

### ST499^Pas^ as a dominant non-CC2 CR*Ab* lineage in the United States.

ST499^Pas^ was the most common non-CC2 lineage in this study, accounting for 16% of all isolates. While ST499 isolates have been found sporadically in the past, prior studies of CR*Ab* specifically have focused on CC1 to CC3. We therefore reviewed our findings in the context of ST499^Pas^ genomes deposited in the NCBI database from a variety of locations (Fig. S1). A group of 11 closely related isolates represented an outbreak of CR*Ab* in a Chicago (IL)-area hospital between 2009 and 2012 (22). Another 14 isolates, most of which were carbapenem resistant, were collected in Maryland in 2011 and 2012 (23). The rest of the isolates in the database were reported from Kentucky, Ohio, and Tanzania. Phylogenetic analysis of all ST499^Pas^ genomes from SNAP and NCBI showed a diverse population, with a median pairwise SNP distance of 1,386 (range, 0 to 7,803) over the 2.3-Mb core genome. A majority of the ST499^Pas^ genomes in the NCBI database were identified as carbapenem resistant in respective reports; however, we were not able to identify previously known carbapenemase genes in 46% (16/35) of isolates. In contrast, all SNAP ST499^Pas^ isolates possessed an acquired carbapenemase gene. Among the combined SNAP and NCBI ST499^Pas^ genomes, we identified three different acquired OXA carbapenemases: *bla*_OXA-24/40_, *bla*_OXA-23_, and *bla*_OXA-72_ (22, 24, 25). The ARLG-6420 RI identified in our cohort was found only in two unrelated NCBI-deposited genomes.

10.1128/mBio.02759-21.9 FIG S1 Core genome phylogeny of ST499^Pas^ isolates from SNAP (*n* = 20) and NCBI (*n* = 35). The midpoint-rooted phylogeny was constructed from SNPs detected in the core genomes of all isolates (2.3-Mb core genome length), using RAxML. Branches are colored by geographical site of isolation, and the colored ring indicates known carbapenemase types. Download FIG S1, TIF file, 0.3 MB. Copyright © 2022 Iovleva et al. 2022 Iovleva et al. https://creativecommons.org/licenses/by/4.0/ This content is distributed under the terms of the Creative Commons Attribution 4.0 International license.

## DISCUSSION

CR*Ab* poses a significant problem worldwide due to its high frequency of multidrug resistance and limited options for effective treatment. In 2019, the CDC Antimicrobial Resistance Threats Report listed CR*Ab* as an urgent public health threat due to limited treatment options and also pointed to its potential to spread carbapenemase genes to non-Acinetobacter health care-associated pathogens (4). Here, we describe the contemporary clinical and genome epidemiology of 120 patients and associated bacterial isolates registered at four major medical centers in the United States.

Patients infected or colonized with CR*Ab* were older and were admitted from health care settings, such as long-term-care facilities. A majority of the patients had comorbid conditions, and almost half of them were critically ill at the time of initial presentation. A majority of CR*Ab* isolates were isolated from respiratory and wound sources.

Most studies of clinical outcomes of CR*Ab* infection have been derived from observational or retrospective studies, often from single hospital systems. Mortality estimates associated with CR*Ab* infection in the past have been highly variable, ranging between 16% and 76% (26). In our study, all-cause 30- and 90-day mortality rates were 24% and 27%, respectively. In patients determined to have infection, both 30- and 90-day mortality was 26%. These findings underscore the idea that CR*Ab* poses a threat to the most vulnerable patients and contributes to high morbidity and mortality. Viewed another way, CR*Ab* appears to colonize and infect patients who are at high risk for poor outcomes.

CC2 was the most prevalent CR*Ab* lineage in our study, followed by ST499^Pas^. The two dominant lineages differed in plasmid content, with CC2 isolates having generally more plasmids than non-CC2 isolates, including ST499^Pas^. Additionally, different genomic regions were affected by recombination in CC2 and ST499^Pas^. Most notably, the CPS locus was a hot spot for recombination in CC2 isolates, indicating possible selection for diversification of this trait but also confounding strain assignments based on the Oxford ST scheme. Once these recombination events were accounted for, sublineages within CC2 and ST499^Pas^ were no longer distinguishable, demonstrating the role of recombination events in their ongoing diversification. The sublineages within CC2 and ST499^Pas^ differed in their geographic distribution, antimicrobial susceptibilities, and plasmid and RI content. Overall, these data demonstrate that recombination as well as plasmid and RI content plays an important role in the emergence and differentiation of CR*Ab* clonal lineages and their acquisition of antimicrobial resistance determinants (27).

In comparison to prior studies conducted in Cleveland and Pittsburgh, we saw a shift in the dominant CR*Ab* sublineages within CC2. A previous molecular epidemiological study of CR*Ab* in the United States from 2008 and 2009 showed predominance of ST2^Pas^/122^Ox^ and ST2^Pas^/208^Ox^ among CR*Ab* isolates in Pittsburgh (6). In the present study, we did not identify any ST2^Pas^/122^Ox^ or ST2^Pas^/208^Ox^ isolates at this study site. Instead, sublineages CC2B (ST2^Pas^/451^Ox^) and CC2C (ST2^Pas^/281^Ox^) were dominant. Similarly, ST2^Pas^/208^Ox^ appears to have been replaced by ST2^Pas^/281^Ox^ in Cleveland hospitals. This is consistent with a recent report by Adams et al. describing replacement of ST208^Ox^ by ST281^Ox^ in two Cleveland health care systems (11). While similar longitudinal data are not available for us to make similar comparisons for other study sites, these findings provide a useful point of reference for identifying changes in CR*Ab* populations going forward. On the other hand, the identification of ST499^Pas^ as a dominant non-CC2 CR*Ab* lineage was unexpected. While ST499^Pas^ isolates have been sporadically reported from different U.S. cities, it has not been generally considered a prevalent lineage in the United States.

On balance, each hospital system in our study appeared to have its own unique CR*Ab* population. The CR*Ab* populations within individual hospitals were similar to those in the health care systems they belonged to. This is not surprising and can be explained by movement of patients and health care workers between hospitals and long-term-care facilities within the same geographical areas. Some sublineages, like CC2C (ST2^Pas^/ST281^Ox^), were widely distributed, while others were more localized, like sublineage CC2B (ST2^Pas^/ST451^Ox^), which was found primarily in Pittsburgh. Generally, in study sites with many CR*Ab* cases, few clonal sublineages dominated, suggesting endemicity within hospitals or associated facilities, as CR*Ab* is known to survive for prolonged periods of time on environmental surfaces such as hospital beds and in sinks and other plumbing (28, 29). While our study was not designed to investigate patient-to-patient transmission, core genome SNP analyses showed that the majority of same-patient, same-sublineage isolates fell within 0 to 10 SNPs of one another. This finding may be useful in the interpretation of genome sequencing data of CR*Ab* in the context of hospital epidemiology.

The antibiotic susceptibility profiles of the CR*Ab* isolates were distinct from those in prior surveys. While different sublineages had distinct profiles, we observed a general trend in decreased resistance to cephalosporins and aminoglycosides in our cohort compared to a prior survey, as well as an increase in nonsusceptibility to ampicillin-sulbactam (6). A concerning finding was the high rate of colistin resistance. The overall colistin resistance rate in our study was 22%, and sublineage CC2C (ST281^Ox^) was the main driver, with nearly 40% of isolates being resistant. This is in contrast with a recent study reporting a colistin resistance rate of 8.7% among meropenem-nonsusceptible A. baumannii isolates collected from hospitals in North America in 2014, as well as the colistin resistance rate of 16.6% reported in a recent study from Europe (30, 31). The fact that we uncovered diverse mutations in *pmrB* and *lpxD* among the colistin-resistant isolates, and that they were interspersed throughout the sublineage CC2C phylogeny, suggests that colistin resistance probably evolved *de novo* in each patient, rather than spreading through transmission of a single resistant clone. It is also possible that sublineage CC2C (ST281^Ox^) is more adept at maintaining colistin resistance, which may have led to overrepresentation of colistin-resistant isolates belonging to this sublineage. This finding has implications for empirical treatment options, since colistin or polymyxin B is still often the mainstay of therapy, and susceptibility testing is delayed given the complexity of current testing strategies. Nonetheless, these findings highlight the need for development of new testing strategies for rapid determination of colistin susceptibility, along with alternative therapies, to minimize the risk of administering inactive therapy.

One novel therapy that could be useful for the treatment of CR*Ab* is cefiderocol, a siderophore cephalosporin approved in 2019, after this study. Approximately 6% of the CR*Ab* isolates were found to be resistant to cefiderocol using CLSI breakpoints, which is comparable with previously reported surveillance data (30). While resistance to cefiderocol was uncommon, recent evidence of higher all-cause mortality in patients who were infected with CR*Ab* and treated with cefiderocol in the CREDIBLE-CR trial (which compared cefiderocol to the best available therapy for carbapenem-resistant organisms) suggests that the role of cefiderocol in the treatment of invasive CR*Ab* infections remains unclear (32). However, it might still be considered as a rescue therapy in critically ill patients, in which case susceptibility of the isolate should be confirmed (33).

This study had several limitations. We included only patients and isolates from four large hospital systems that were the initial participants of the SNAP cohort. These study sites were largely self-selected and might not be representative of the U.S. population as a whole. Also, this study was restricted in time to 12 months and was thus unable to examine changes in CR*Ab* populations over time. Furthermore, the sample size was relatively small, which makes it difficult to draw clinically relevant conclusions regarding lineage-specific antimicrobial susceptibility trends and how they affect isolates’ responses to antimicrobial therapy. Finally, this was not designed as transmission study; thus, while we are able to hypothesize possible transmission events by using a cutoff of 10 SNPs (maximum amount of core genome SNPs [cgSNPs] found in the same patient), we do not have associated hospital epidemiology data to infer possible intra- or interfacility transmission events.

We expect to address many of these limitations in future studies of the full SNAP data set. Systematic studies of CR*Ab* are of paramount importance for devising strategies to prevent their dissemination and improve clinical outcomes. The full SNAP data set will provide a robust resource for studying CR*Ab* biology and associated clinical outcomes in a systematic, clinically informed manner.

Overall, our findings highlight the continued importance of CR*Ab* as a health care-associated pathogen causing high morbidity and mortality and with limited treatment options, as well as the significance of real-time surveillance and genomic epidemiology in studying its dissemination and clinical impact.

## MATERIALS AND METHODS

### Patients.

Patients were included in the study if CR*Ab* was isolated in a clinical culture from any anatomic site during hospitalization between September 2017 and October 2018. Surveillance cultures were not included. A total of 23 hospitals in four quaternary health systems (Cleveland Clinic Foundation, University of Pittsburgh Medical Center, University of Texas Health Science Center at Houston, and University of North Carolina Chapel Hill) enrolled patients in this study phase. The study was approved by the institutional review boards (IRB) of all the health systems with a waiver of patient consent.

### Clinical information.

Clinical data collected from electronic health records included patient demographics, underlying comorbidities (Charlson comorbidity index [CCI]), severity of illness as defined by the Pitt bacteremia score, microbiology reports, resolution of infection symptoms, duration of hospital stay, disposition after discharge, readmission at 90 days, mortality at 30 and 90 days, and infection versus colonization status (8, 34). Infection and colonization were defined by previously described criteria, with the exception of respiratory infections, as patients with CR*Ab* respiratory infections do not necessarily meet the criteria outlined by the American Thoracic Society and the Infectious Diseases Society of America (35–37). Respiratory isolates were considered to cause an infection if the respiratory diagnosis on the case report form was tracheobronchitis, pneumonia without mechanical ventilation, ventilator-associated pneumonia, or an “other” diagnosis after review by two study investigators. All other cultures, including those missing information needed for the assignment of infection/colonization, were considered to represent colonization. The DOOR (desirability of outcome ranking) analysis was used to assess the following deleterious and adverse events: (i) absence of clinical and symptomatic response or relapse of infection; (ii) unsuccessful discharge, which included death, discharge to hospice, hospitalization for >30 days, and readmission within 30 days; (iii) new-onset renal failure within 30 days after the index culture; and (iv) Clostridioides difficile infection within 30 days after index culture, as described previously (35).

### Microbiology.

Bacterial identification and susceptibility testing were performed by each contributing microbiology laboratory using Biotyper (Bruker, Billerica, MA, USA), MicroScan (Beckman Coulter, Atlanta, GA, USA), or VitekMS, Vitek2, or Etest (all bioMérieux, Durham, NC, USA), BD Phoenix or BBL disks (both BD, Durham, NC, USA), Sensititre (Thermo Fisher, Waltham, MA, USA), or disk diffusion methods. Carbapenem resistance was determined based on the Clinical and Laboratory Standards Institute (CLSI) interpretive criteria for meropenem or imipenem nonsusceptibility (MIC, ≥4 mcg/ml).

At the central research laboratory, MICs of each agent active against A. baumannii (amikacin, gentamicin, tobramycin, doxycycline, minocycline, tigecycline, ciprofloxacin, levofloxacin, trimethoprim-sulfamethoxazole, imipenem, meropenem, doripenem, cefepime, ceftazidime, ampicillin-sulbactam, and colistin) were determined using Sensititre GNX3F lyophilized plates (Thermo Fisher Scientific, Waltham, MA). CLSI breakpoints were used to determine susceptibility. Only one CR*Ab* isolate was identified as carbapenem susceptible.

Cefiderocol susceptibility testing was performed using an iron-depleted, cation-adjusted Mueller-Hinton broth microdilution panel (International Health Management Associates, Schaumburg, IL, USA). Cefiderocol MIC results were interpreted using CLSI-approved breakpoints (susceptible [S], 4 μg/mL; intermediate [I], 8 μg/mL; resistant [R], 16 μg/mL) (38). As CLSI breakpoints are not available for tigecycline, we defined susceptibility as a MIC of ≤2 μg/mL and nonsusceptibility as a MIC of ≥4 μg/mL, based on previous literature and Food and Drug Administration (FDA) breakpoints for *Enterobacterales* (S, 2 μg/mL; I, 4 μg/mL; R, 8 μg/mL) (39, 40).

### Whole-genome sequencing and phylogenetic analysis.

Genomic DNA was extracted from isolates using a DNeasy blood and tissue kit (Qiagen, Germantown, MD). Whole-genome sequencing was performed on a NextSeq 550 instrument (Illumina, San Diego, CA), using 2 × 150-bp paired-end reads, at the Microbial Genome Sequencing Center (Pittsburgh, PA). Additionally, five isolates representing major sublineages were sequenced with long-read technology on an Oxford Nanopore MinION device (Oxford Nanopore Technologies, Oxford, United Kingdom). Resulting reads were quality processed through our bioinformatics pipeline. Five isolates were excluded from further molecular and antimicrobial susceptibility analysis, as they were identified as bacterial species other than A. baumannii (4 isolates) or were carbapenem-susceptible A. baumannii (1 isolate). Details of sequencing, bioinformatics, and phylogenetic analyses are available in Text S1.

10.1128/mBio.02759-21.1 TEXT S1 Details of sequencing, bioinformatics, and phylogenetic analyses. Download Text S1, DOCX file, 0.02 MB. Copyright © 2022 Iovleva et al. 2022 Iovleva et al. https://creativecommons.org/licenses/by/4.0/ This content is distributed under the terms of the Creative Commons Attribution 4.0 International license.

### Statistics.

The chi-square and Fisher’s exact tests were used as appropriate to compare antimicrobial susceptibility trends between lineages/sublineages. The Mann-Whitney test was used to compare number of plasmids and *repA* genes between lineages.

### Data availability.

Raw sequence reads and draft genome assemblies have been deposited in the NCBI database under BioProject number PRJNA667103, accession numbers SAMN16351076 to SAMN16351208.
