# Supplementary material for: Carbapenem-Resistant Acinetobacter baumannii in U.S. Hospitals: Diversification of Circulating Lineages and Antimicrobial Resistance
Source: mBio. 2022 Mar 21;13(2):e02759-21. doi: 10.1128/mbio.02759-21 (PMC9040734; doi:10.1128/mbio.02759-21)
Supplement: TABLE S1 [file mbio.02759-21-st001.docx]

**Supplementary Table 1.** Pairwise core genome SNP comparisons calculated from total core genome of 150 isolates from 120 patients included in the study.

| **Lineage < 10,000 SNPs** | **n** | **Core genome length** | **median SNPs**  **(range)** |
| --- | --- | --- | --- |
| CC2 (ST2^Pas^) | 119 | 2,602,279 | 930 (0-8060) |
| CC499 (ST499^Pas^) | 20 |  | 1,289 (4-5211) |
| ST46^Pas^ | 2 |  | 47 (N/A**) |
| ST79^Pas^ | 2 |  | 873 (N/A) |
| ST229^Pas^ | 1 |  | N/A |
| ST1088^Pas^-like* | 6 |  | 7,328 (3-7,608) |

*ST1088-like includes isolates belonging to ST1088, ST1091, and a new ST pending assignment.

**N/A, not applicable, too few isolates for calculation.
