# Supplementary material for: Carbapenem-Resistant Acinetobacter baumannii in U.S. Hospitals: Diversification of Circulating Lineages and Antimicrobial Resistance
Source: mBio. 2022 Mar 21;13(2):e02759-21. doi: 10.1128/mbio.02759-21 (PMC9040734; doi:10.1128/mbio.02759-21)
Supplement: TABLE S2 [file mbio.02759-21-st002.docx]

**Supplementary Table 2.** Pairwise core genome SNP comparisons among major sub-lineages calculated from total core genome of 150 CR*Ab* isolates from 120 study patients.

| **Lineage <10,000 SNPs** | **Sub-lineage <2,000 SNPs** | **Proportion of core genome** | **Included STs**  **(Oxford)** | **n** | **median SNPs**  **(range)** |
| --- | --- | --- | --- | --- | --- |
| CC2 | A | 76% | 208 | 11 | 834 (0-2,220) |
|  |  |  | 417 | 3 |  |
|  |  |  | 218 | 2 |  |
|  | B | 92% | 451 | 18 | 18 (0-47) |
|  |  |  | 451-SLV* | 2 |  |
|  | C | 76% | 281 | 77 | 99 (1-1,530) |
|  |  |  | 281-SLV* | 4 |  |
|  |  |  | 1899 | 1 |  |
| ST499^Pas^ | D | 87% | 345 | 17 | 965 (4-2,427) |
|  | E |  |  | 3 | 116 (23-125) |

*SLV, single locus variant.
