# Supplementary material for: Carbapenem-Resistant Acinetobacter baumannii in U.S. Hospitals: Diversification of Circulating Lineages and Antimicrobial Resistance
Source: mBio. 2022 Mar 21;13(2):e02759-21. doi: 10.1128/mbio.02759-21 (PMC9040734; doi:10.1128/mbio.02759-21)
Supplement: TABLE S3 [file mbio.02759-21-st003.docx]

**Supplementary Table 3.** Antimicrobial susceptibility profiles of CR*Ab* isolates. Antimicrobial susceptibilities of 115 initial patient isolates were determined with Sensititre plates or broth microdilution. COL, colistin; FDC, cefiderocol; FEP, cefepime; TAZ, ceftazidime; A/S2, ampicillin-sulbactam; TGC, tigecycline; MIN, minocycline; DOX, doxycycline; GEN, gentamicin; AMI, amikacin. S, susceptible; I, intermediate; R, resistant. Susceptibilities were assigned according to the Clinical and Laboratory Standards Institute (CLSI) guidelines. Tigecycline susceptibility was assigned based on FDA *Enterobacterales* breakpoints.

| **Antimicrobial** | **All isolates (%)** | | | **CC2A (%)** | | | **CC2B (%)** | | | **CC2C (%)** | | | **ST499 (%)** | | | **Other (%)** | | |
| --- | --- | --- | --- | --- | --- | --- | --- | --- | --- | --- | --- | --- | --- | --- | --- | --- | --- | --- |
|  | **n=115** | | | **n=13** | | | **n=15** | | | **n=60** | | | **n=18** | | | **n=9** | | |
|  | **S** | **I** | **R** | **S** | **I** | **R** | **S** | **I** | **R** | **S** | **I** | **R** | **S** | **I** | **R** | **S** | **I** | **R** |
| **AMI** | 52.2 | 11.3 | 36.5 | 46.2 | 7.7 | 46.2 | 13.3 | 0.0 | 86.7 | 43.3 | 18.3 | 38.3 | 94.4 | 5.6 | 0.0 | 100.0 | 0.0 | 0.0 |
| **GEN** | 30.4 | 12.2 | 57.4 | 15.4 | 15.4 | 69.2 | 6.7 | 0.0 | 93.3 | 28.3 | 10.0 | 61.7 | 66.7 | 16.7 | 16.7 | 33.3 | 33.3 | 33.3 |
| **DOX** | 66.1 | 4.3 | 29.6 | 0.0 | 0.0 | 100.0 | 46.7 | 0.0 | 53.3 | 91.7 | 3.3 | 5.0 | 38.9 | 16.7 | 44.4 | 77.8 |  | 22.2 |
| **MIN** | 91.3 | 4.3 | 4.3 | 61.5 | 23.1 | 15.4 | 93.3 | 0.0 | 6.7 | 98.3 | 0.0 | 1.7 | 88.9 | 5.6 | 5.6 | 88.9 | 11.1 | 0.0 |
| **TGC** | 98 | 0.0 | 2 | 100.0 | 0.0 | 0.0 | 100.0 | 0.0 | 0.0 | 96.7 | 0.0 | 3.3 | 100.0 | 0.0 | 0.0 | 100.0 | 0.0 | 0.0 |
| **A/S2** | 24.3 | 41.7 | 33.9 | 15.4 | 23.1 | 61.5 | 13.3 | 33.3 | 53.3 | 28.3 | 46.7 | 23.3 | 16.7 | 44.4 | 38.9 | 55.6 | 44.4 | 0.0 |
| **TAZ** | 19.1 | 1.7 | 79.1 | 30.8 | 0.0 | 69.2 | 0.0 | 0.0 | 100.0 | 0.0 | 0.0 | 100.0 | 83.3 | 11.1 | 5.6 | 33.3 | 0.0 | 66.7 |
| **FEP** | 40.0 | 22.6 | 37.4 | 23.1 | 15.4 | 61.5 | 6.7 | 6.7 | 86.7 | 50.0 | 30.0 | 20.0 | 44.4 | 16.7 | 38.9 | 55.6 | 22.2 | 22.2 |
| **FDC** | 94.0 | 0.0 | 6.0 | 100.0 | 0.0 | 0.0 | 93.3 | 0.0 | 6.7 | 90.0 | 0.0 | 10.0 | 100.0 | 0.0 | 0.0 | 88.9 | 0.0 | 11.1 |
| **COL** | N/A | 78.3 | 21.7 | N/A | 92.3 | 7.7 | N/A | 93.3 | 6.7 | N/A | 61.7 | 38.3 | N/A | 100.0 | 0.0 | N/A | 88.9 | 11.1 |
