# Supplementary material for: Carbapenem-Resistant Acinetobacter baumannii in U.S. Hospitals: Diversification of Circulating Lineages and Antimicrobial Resistance
Source: mBio. 2022 Mar 21;13(2):e02759-21. doi: 10.1128/mbio.02759-21 (PMC9040734; doi:10.1128/mbio.02759-21)
Supplement: TABLE S4 [file mbio.02759-21-st004.docx]

**Supplementary Table 4.** CR*Ab* plasmid characteristics.

| **Plasmid name** | **Size, bp** | **Resistance genes** | ***repA* group** |
| --- | --- | --- | --- |
| ARLG-6295_4 | 10,967 | none | GR20 |
| ARLG-6376_3 | 11,194 | none | GR8 |
| ARLG-6420_2 | 11,323 | *bla*_OXA-207_ | GR2 |
| ARLG-6344_3 | 68,153 | none | GR32 |
| ARLG-6344_2 | 73,194 | *bla*_OXA-23_ | GR6 |
| ARLG-6295_2 | 166,694 | *bla*_OXA-23_ *aphA6* | GR6, GR32 |
