# Supplementary material for: Carbapenem-Resistant Acinetobacter baumannii in U.S. Hospitals: Diversification of Circulating Lineages and Antimicrobial Resistance
Source: mBio. 2022 Mar 21;13(2):e02759-21. doi: 10.1128/mbio.02759-21 (PMC9040734; doi:10.1128/mbio.02759-21)
Supplement: TABLE S6 [file mbio.02759-21-st006.docx]

**Supplementary Table 6.** Post ClonalFrameML pairwise core genome SNP comparisons calculated from total core genome of 150 isolates from 120 patients included in the study.

| **Lineages <1,000 SNPs** | **n** | **Core genome length** | **median SNPs (range)** |
| --- | --- | --- | --- |
| CC2 (ST2^Pas^) | 118 | 2,602,279 | 29 (0-120) |
| CC499 (ST499^Pas^) | 20 |  | 14.5 (0-48) |
| ST46^Pas^ | 2 |  | 10 (N/A*) |
| ST79^Pas^ | 2 |  | 24 (N/A) |
| ST229^Pas^ | 1 |  |  |
| ST1088^Pas^-like | 6 |  | 311 (0-318) |

*N/A, not applicable, too few isolates for calculation.
