# Supplementary material for: Carbapenem-Resistant Acinetobacter baumannii in U.S. Hospitals: Diversification of Circulating Lineages and Antimicrobial Resistance
Source: mBio. 2022 Mar 21;13(2):e02759-21. doi: 10.1128/mbio.02759-21 (PMC9040734; doi:10.1128/mbio.02759-21)
Supplement: TABLE S7 [file mbio.02759-21-st007.docx]

**Supplementary Table 7.** Post ClonalFrameML pairwise core genome SNP comparisons among major sub-lineages calculated from total core genome of 150 CR*Ab* isolates from 120 study patients.

| **Lineages <1,000 SNPs** | **Sub-lineages <60 SNPs** | **Included STs**  **(Oxford)** | **n** | **median SNPs (range)** |
| --- | --- | --- | --- | --- |
| CC2 (ST2^Pas^) | A | 208 | 11 | 26 (0-46) |
|  |  | 417 | 3 |  |
|  |  | 218 | 2 |  |
|  |  | 451 | 18 |  |
|  |  | 451-SLV | 2 |  |
|  | B | 281 | 77 | 16 (0-50) |
|  |  | 281-SLV | 4 |  |
|  |  | 1899 | 1 |  |
| ST499^Pas^ |  | 345 | 20 | 15 (0-48) |
