# Supplementary material for: Carbapenem-Resistant Acinetobacter baumannii in U.S. Hospitals: Diversification of Circulating Lineages and Antimicrobial Resistance
Source: mBio. 2022 Mar 21;13(2):e02759-21. doi: 10.1128/mbio.02759-21 (PMC9040734; doi:10.1128/mbio.02759-21)
Supplement: TABLE S5 [file mbio.02759-21-st005.docx]

**Supplementary Table 5.** Sub-lineage specific pairwise non-recombinant SNP comparisons between isolates sampled from the same patient, the same study site, and different study sites calculated from total core genome of 150 CR*Ab* isolates from 120 patients.

| **Sub-lineages** | **n** | **Same patient SNPs** | **Same hospital SNPs** | **Same site SNPs** | **Different site SNPs** |  |
| --- | --- | --- | --- | --- | --- | --- |
|  |  | **median (range)** | **median (range)** | **median (range)** | **median (range)** |  |
| CC2A | 16 | 0 (NA) | 25 (11-35) | 23 (0-38) | 27 (1-44) |  |
| CC2B | 20 | 1 (0-3) | 8 (0-12) | 7 (0-13) | 9 (0-15) |  |
| CC2C | 82 | 1 (1-10) | 13 (0-50) | 14 (0-43) | 21 (0-50) |  |
| ST499^Pas^ D | 17 | 0 (NA) | 4 (0-20) | 9 (1-22) | 14(11-24) |  |
| ST499^Pas^ E | 3 | N/A* | 8 (N/A) | 5 (1-22) | N/A |  |
| All same sub-lineage |  | 0 (0-10) | 13 (0-50) | 14 (0-43) | 20 (0-50) |  |

*N/A, not applicable, too few isolates for calculation.
