# Supplementary figures and images for: Carbapenem-Resistant Acinetobacter baumannii in U.S. Hospitals: Diversification of Circulating Lineages and Antimicrobial Resistance
Source: mBio. 2022 Mar 21;13(2):e02759-21. doi: 10.1128/mbio.02759-21 (PMC9040734; doi:10.1128/mbio.02759-21)

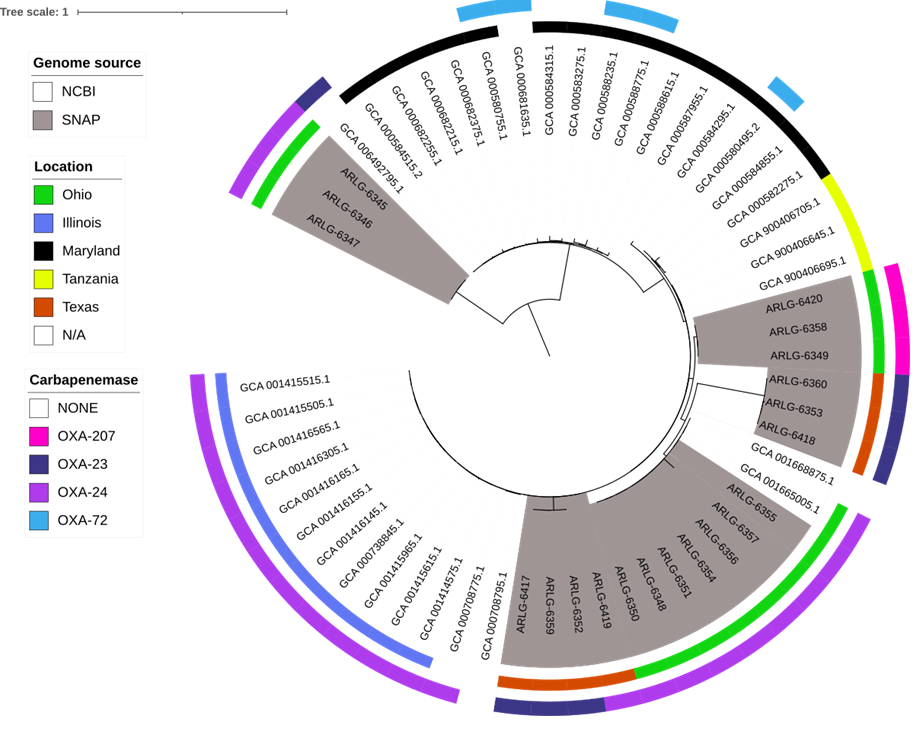

Supplement: FIG S1 [file mbio.02759-21-sf001.tif]
