# Supplementary material for: Carbapenem-Resistant Acinetobacter baumannii in U.S. Hospitals: Diversification of Circulating Lineages and Antimicrobial Resistance
Source: mBio. 2022 Mar 21;13(2):e02759-21. doi: 10.1128/mbio.02759-21 (PMC9040734; doi:10.1128/mbio.02759-21)
Supplement: TEXT S1 [file mbio.02759-21-s0001.docx]

**Whole genome sequencing.**

Genomic DNA was extracted from isolates using a DNeasy Blood & Tissue Kit (Qiagen, Germantown, MD). Whole genome sequencing was performed on a NextSeq 550 instrument (Illumina, San Diego, CA), using 2x150-bp paired-end reads at the Microbial Genome Sequencing Center (Pittsburgh, PA). Illumina sequence reads were assembled using SPAdes v3.13. Genome assemblies were required to meet the following criteria to be included in the analysis: (i) average read depth greater than 40x; (ii) genome length between 3.2-4.8 Mb; (iii) total number of contigs below 350; and (iv) N50 value greater than 45 kb. Additionally, all assembled genomes were subjected to Kraken (v1) taxonomic sequence classification for species identification and to rule out contamination

Five isolates were sequenced with long-read technology on an Oxford Nanopore MinION device (Oxford Nanopore Technologies, Oxford, United Kingdom). The long-read sequencing library was prepared and multiplexed using a rapid multiplex barcoding kit (catalog SQK-RBK004) and was sequenced on a R9.4.1 flow cell. Base-calling on raw reads was performed using Guppy v2.3.1 (Oxford Nanopore Technologies). Hybrid assembly for each of these isolates was conducted using unicycler v0.4.8-beta, using long MinION reads and short Illumina reads from the same isolate.

**Phylogenetic analysis.**

Genetic variants were identified by mapping raw Illumina sequencing reads from each isolate to a previously generated, closely related, high-quality closed reference genome of strain S1 (CP026943.1). Single-nucleotide polymorphism (SNP) differences between genome pairs were identified using Snippy v4.4.5 (<https://github.com/tseemann/snippy>), with complex variants greater than 10 nucleotides filtered out of the freebayes output. Recombinant SNPs were identified and removed using ClonalFrameML 1.11. Sequence types were determined *in silico* using the online Pasteur(^Pas^) and Oxford(^Ox^) multilocus sequence typing (MLST) databases. A phylogenetic tree based on SNPs in core genes was reconstructed with RAxML v8.2.11 using the general time-reversible model of evolution, Γ-distributed rate variation among sites (GTRGAMMA) and 100 rapid bootstrap replicates and was visualized and annotated using iTOL v4. SNP cutoffs that correlate with Pasteur and Oxford sequence types (STs) were defined by comparing pairwise SNP differences between all 150 study isolates before and after ClonalFrameML analysis. Prophage regions and mobile genetic elements (MGEs) were identified using PHASTER and MobileElementFinder.

Genetic diversity within and between patients for isolates belonging to the same sub-lineage was examined by comparing non-recombinant pairwise SNP distances in four different epidemiologic categories: *same patient*, *same hospital,* *same study site*, and *different study site*.

To construct a phylogenetic tree specific to lineage ST499^Pas^, 35 available ST499^Pas^ assembled genomes were downloaded from NCBI GenBank on August 20, 2019. An alignment of core genes was generated using Roary version 3.12.0. A phylogenetic tree of all GenBank and SNAP ST499^Pas^ genomes was built with RAxML with the generalized time-reversible model of evolution, a categorical model of rate heterogeneity (GTR-CAT) and 100 bootstrap replicates.

**Analysis of plasmids and resistance islands.**

We randomly selected 1-2 representative isolates from each lineage for long-read sequencing with MinION. Hybrid genome assemblies were created, and closed, 6 circular plasmids were identified from these. Subsequently, contigs from Illumina assemblies from all other isolates were mapped to these plasmid sequences using BLASTn, as described previously. A plasmid was determined to be present in an isolate if at least 90% of the gene content of the reference plasmid was present at 95% sequence identity or greater. Contigs were also examined for the presence of replication initiation protein families using BLASTn against previously reported *repA* sequences. We retained hits with at least 95% sequence identity.

The presence of previously described genomic resistance islands (RIs) was determined by BLASTn. Genomic contigs from isolates were mapped against the following previously characterized RIs: AbGRI1, AbGRI2, AbGRI3, and AbGRI4. The conservation of reference RI gene content was calculated by percent mapping using BLASTn. RI was deemed to be present if at least 70% of the reference RI gene content was observed. The new resistance island in ST499^Pas^ isolate ARLG-6420 was defined using BLASTn and ISFinder.

**Resistance gene identification.**

ResFinder and NCBI’s Bacterial Antimicrobial Resistance Reference Gene databases were queried using BLASTn with an 80% sequence identity cut-off to identify antimicrobial resistance genes. Results of BLASTn queries and sequence alignments for genes associated with colistin resistance (*pmrCAB*, *lpxA*, *lpxC*, and *lpxD*) were manually curated to identify specific point mutations.
